# Spatiotemporal Dynamic Regulation of Organelles During Meiotic Development, Insights From Fungi

**DOI:** 10.3389/fcell.2022.886710

**Published:** 2022-04-25

**Authors:** Fernando Hernández-Sánchez, Leonardo Peraza-Reyes

**Affiliations:** Departamento de Bioquímica y Biología Estructural, Instituto de Fisiología Celular, Universidad Nacional Autónoma de México, Mexico City, Mexico

**Keywords:** endoplasmic reticulum, peroxisome, nuclear envelope, mitochondria, organelle dynamics, development, meiosis, fungi

## Abstract

Eukaryotic cell development involves precise regulation of organelle activity and dynamics, which adapt the cell architecture and metabolism to the changing developmental requirements. Research in various fungal model organisms has disclosed that meiotic development involves precise spatiotemporal regulation of the formation and dynamics of distinct intracellular membrane compartments, including peroxisomes, mitochondria and distinct domains of the endoplasmic reticulum, comprising its peripheral domains and the nuclear envelope. This developmental regulation implicates changes in the constitution and dynamics of these organelles, which modulate their structure, abundance and distribution. Furthermore, selective degradation systems allow timely organelle removal at defined meiotic stages, and regulated interactions between membrane compartments support meiotic-regulated organelle dynamics. This dynamic organelle remodeling is implicated in conducting organelle segregation during meiotic differentiation, and defines quality control regulatory systems safeguarding the inheritance of functional membrane compartments, promoting meiotic cell rejuvenation. Moreover, organelle remodeling is important for proper activity of the cytoskeletal system conducting meiotic nucleus segregation, as well as for meiotic differentiation. The orchestrated regulation of organelle dynamics has a determinant contribution in the formation of the renewed genetically-diverse offspring of meiosis.

## Introduction

Sexual reproduction enables eukaryotic organisms to produce genetically diverse offspring. In this process, haploid reproductive cells produced by different individuals fuse to generate a diploid, whose genomic content is later converted back to haploid, enabling subsequent reproductive cycles. Central to this process is meiosis, a specialized division that reduces diploid genome by half, while enabling genetic recombination. Meiosis requires precise regulation between defined cellular architectural changes and nuclear progression, and is finely coordinated with the differentiation of the cells carrying the meiotic nuclear products. Ultimately, meiotic differentiation promotes cell functioning renewal and progeny rejuvenation (Unal and Amon, 2011; Unal et al., 2011).

**FIGURE 1:**
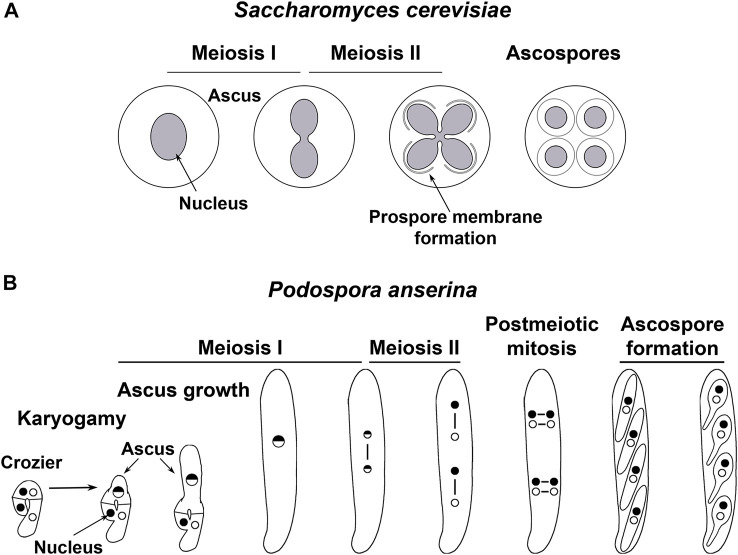
Meiotic development in two model fungi. **(A)** In the budding yeast *Saccharomyces cerevisiae*, diploid cells produced after mating undergo meiosis when exposed to nutritional limitations. During this process, the NE remains continuous along both meiotic divisions. By meiosis II, new membranes are produced from the NE-embedded SPBs (not depicted) of each lobe of the dividing nucleus–termed prospore membranes–, which expand encircling each nucleus and a fraction of cytoplasmic contents. Meiotic spores (ascospores) are delineated upon closure of the prospore membrane, which separates the ascospore cytoplasm from the cytoplasm of the original mother cell. Ultimately, proteases released after permeabilization of the mother cell vacuole degrade the cytoplasmic contents excluded from ascospores (Neiman, 2011; Eastwood et al., 2012; Eastwood and Meneghini, 2015). **(B)**. In the mycelial ascomycete *Podospora anserina*, sexual development takes place within multicellular fructifications known as perithecia (not shown). Within these structures, nuclei from both parental origins (depicted as dots with different shading) are isolated in pairs in specialized cells termed croziers. The crozier dikaryotic cell undergoes karyogamy while enters meiosis and differentiates into an ascus (the meiocyte), which elongates along prophase I. After meiosis completion, the four resulting nuclei divide mitotically to yield eight nuclei, which are packaged by pairs into four ascospores (Zickler et al., 1995).

Studies conducted in fungi have provided essential knowledge about the developmental processes of sexual reproduction, including karyogamy (Rose, 1996; Gibeaux and Knop, 2013) and meiosis (e.g., Marston and Amon, 2004; Zickler and Espagne, 2016; Grey and de Massy, 2021; Sato et al., 2021). Fungal meiotic differentiation often culminates with the formation of haploid meiotic spores (Figure 1). This process is equivalent to gametogenesis and has provided valuable knowledge about it (van Werven and Amon, 2011; Anandhakumar et al., 2013; Goodman et al., 2020). Research with different fungi has recently disclosed that meiotic development involves sophisticated spatiotemporal remodeling of multiple organelles, which is critically for sexual development.

### Peroxisome Dynamics and Assembly are Regulated During Meiotic Development

Peroxisomes are versatile and highly dynamic organelles that perform multiple metabolic functions–including prominent roles in lipid and redox metabolism–and are involved in diverse cellular processes, like cell signaling (Deb and Nagotu, 2017). Research in fungi provided initial evidence that peroxisome dynamics are regulated during meiotic development (Berteaux-Lecellier et al., 1995). In the mycelial fungus *Podospora anserina* (Figure 1), peroxisomes proliferate during meiocyte (ascus) and meiotic-spore (ascospore) differentiation, whereas their number decreases during ascospore maturation. Meiotic development also involves changes in peroxisome distribution and morphology, including peroxisome accumulation at the ascus growing apical region during its differentiation, and elongation from meiosis II until ascospore differentiation (Berteaux-Lecellier et al., 1995; Takano-Rojas et al., 2016, reviewed in; Navarro-Espíndola et al., 2020a). Meiotic peroxisome remodeling correlates with changes in the functional state of the protein machinery that transports proteins into peroxisomes (Peraza-Reyes et al., 2011), suggesting concerted developmental regulation of peroxisome dynamics and biogenesis. This regulation involves distinct configurations of the peroxisome protein import pathways, which are respectively required for karyogamy and meiotic initiation (Berteaux-Lecellier et al., 1995; Peraza-Reyes et al., 2011; Suaste-Olmos et al., 2018), and for proper meiotic progression (Bonnet et al., 2006). Peroxisome remodeling also involves the activity of the dynamin-related GTPase Dnm1, and its membrane anchor Fis1. These proteins drive peroxisome fission throughout sexual development and promote peroxisome segregation during ascus and ascospore differentiation (Navarro-Espindola et al., 2020b). Fis1 and Dnm1 are required for timely karyogamy and proper ascospore differentiation; still, as they also mediate mitochondrial fission (Navarro-Espindola et al., 2020b) (see below), their precise contribution to these processes awaits elucidation. Nonetheless, these findings underscore the close interrelationship of the mechanisms used by cells to regulate organelle dynamics, and disclose their relevance for meiotic development.

In *P. anserina*, peroxisome removal from late ascospores could be mediated by selective autophagy (pexophagy) and represent a quality control mechanism involved in ascospore rejuvenation (Takano-Rojas et al., 2016). Consistently, elimination of Atg24 –a sorting nexin involved in pexophagy, mitophagy and non-selective autophagy–reduces the lifespan of this fungus (Henkel et al., 2020). Furthermore, in the mycelial ascomycete *Sordaria macrospora*, loss of the pexophagy receptor NBR1 (Werner et al., 2019), or of the peroxisomal protease LON2 (Werner et al., 2021), decreases ascospore formation, suggesting parallel systems for peroxisome quality control during ascospore formation. Still, in *Saccharomyces cerevisiae*, peroxisomes are segregated meiotically, but changes in their intracellular dynamics, at least concerning their abundance, are less apparent (Gurvitz et al., 1998), suggesting different requirements for peroxisome dynamics in distinct meiotic developmental processes.

To understand the meiotic involvement of peroxisomes it will be critical to elucidate the configuration of the peroxisome import pathways along meiotic development–including the identification of their imported proteins–, and to disclose how their regulation is linked to the control of peroxisome dynamics. In turn, understanding whether these processes are connected to the regulation of the dynamics of other organelles will shed light into how peroxisomes integrate into the developmental intracellular dynamics leading meiosis.

### Spatiotemporal Remodeling of Mitochondria During Meiotic Development

Early observations in *S. cerevisiae* showed that mitochondria change their distribution during meiosis by concentrating around the nucleus by meiosis II (Zickler and Olson, 1975). Further detailed analyses showed that changes in mitochondria distribution are accompanied by remodeling by fusion and fission, where fragmented mitochondria present in pre-meiotic cells fuse upon meiotic initiation, and the resulting elongated mitochondria move to the nuclear periphery by the second meiotic division. Mitochondria remain associated with nuclei until ascospore formation, which facilitates their incorporation into nascent ascospores. Finally, mitochondria detach from the nuclear envelope (NE) and fragment again in late ascospores (Miyakawa et al., 1984; Gorsich and Shaw, 2004; Suda et al., 2007).

Critical for the positional control of mitochondria during meiotic differentiation is the precise developmental control of mitochondria tethering. In *S. cerevisiae*, early meiotic cells retain mitochondria at the cell cortex through interactions with the mitochondria–endoplasmic reticulum (ER)–cortex anchor (MECA) (Sawyer et al., 2019), a complex composed of proteins Num1 and Mdm36 that attaches mitochondria and the ER at the plasma membrane (Cerveny et al., 2007; Klecker et al., 2013; Lackner et al., 2013). At the onset of anaphase II, the meiotic transcriptional regulator Ndt80 activates the meiosis-specific kinase Ime2, which phosphorylates MECA and promotes its degradation. This facilitates the release of mitochondria from the plasma membrane and allows their subsequent packaging into ascospores (Sawyer et al., 2019). In the fission yeast *Schizosaccharomyces pombe*, equivalent interactions by the Num1 orthologue Mcp5 –a meiosis-specific protein in this yeast–tether mitochondria to the plasma membrane of meiotic cells (Saito et al., 2006; Yamashita and Yamamoto, 2006; Chacko et al., 2019; Kraft and Lackner, 2019). However, in this yeast, mitochondria of opposite parental origin are retained at opposed ends of meiotic cells, which promotes their uniparental meiotic inheritance (Chacko et al., 2019). In *S. cerevisiae*, a second tether presumably attaches mitochondria to the NE by meiosis II, as inferred by their close apposition to nuclei (Zickler and Olson, 1975; Suda et al., 2007; Sawyer et al., 2019). This tether could conceivably ensure proper meiotic mitochondrion partitioning and participate in a quality control system determining the inheritance of healthy mitochondria (Sawyer et al., 2019). The identification of such a tether could be critical to understand the cellular basis of meiotic rejuvenation.

Proper mitochondrion inheritance during *S. cerevisiae* meiotic differentiation depends on the fission proteins Dnm1, Mdv1, and Fis1 (Gorsich and Shaw, 2004). In contrast, elimination of Dnm1 or Fis1 does not prevent mitochondria segregation into ascospores in *P. anserina* (Navarro-Espindola et al., 2020b). Moreover, mitochondria fragmentation in *S. cerevisiae* late ascospores occurs in absence of these fission proteins (Gorsich and Shaw, 2004). These observations underscore the different systems involved in the meiotic segregation of mitochondria in distinct organisms and indicate that Dnm1-independent mitochondrial fission processes operate during meiotic differentiation. In *S. cerevisiae*, mitochondrion segregation into ascospores also depends on Ady3, a protein involved in the formation of the ascospore-delineating membrane (the prospore membrane) (Suda et al., 2007), showing that this process contributes to mitochondria partitioning. It is worth highlighting that while the meiotic inheritance of peroxisomes in *P. anserina* depends on Fis1 and Dnm1, that of mitochondria does not (Navarro-Espindola et al., 2020b). This observation discloses different constrains for the segregation of different organelles despite sharing central regulatory factors, and suggests that robust systems stringently control the inheritance of mitochondria, which, unlike peroxisomes, cannot be produced *de novo*. Further research should decipher the molecular mechanisms underlying these systems.

Loss of the fission proteins in *P. anserina* produces severe mitochondrion arrangement alterations in numerous ascospores, suggesting asymmetrical inheritance of mitochondria with different functional states (Navarro-Espindola et al., 2020b). Notably, loss of the fission proteins results in extended lifespan both in *P. anserina* and in mitotically-replicating *S. cerevisiae* (Scheckhuber et al., 2007; Lefevre et al., 2015), indicating a central role for these proteins in regulating cellular lifespan. Nonetheless, further studies are required to understand their precise involvement in regulating ascospore mitochondrial-fitness and rejuvenation, as well as on the peroxisome-mitochondrion interplay during this process. In addition, the mitochondrion-peroxisome fission proteins are required for ascospore differentiation in *P. anserina*, at an early developmental step (Navarro-Espindola et al., 2020b), indicating additional roles for these proteins in meiotic differentiation, beyond organelle segregation and quality control.

### Spatiotemporal Meiotic Remodeling of the ER, With Incursions Into the Nucleus

The ER consists of a continuous membrane system composed of different structural and functional domains, including the NE and distinct peripheral domains (Westrate et al., 2015). As such, the developmental adjustments of these domains are intertwined. A fine-tunned structure-distribution relationship is crucial for proper ER function and quality control.

The NE is composed of the inner and outer nuclear membranes (INM and ONM, respectively), which are continuous at the site of insertion of the nuclear pore complexes (NPCs) and, in fungi, of the spindle pole bodies (SPBs, the NE-embedded fungal equivalents of centrosomes). In turn, the ONM is continuous with the peripheral ER (Ungricht and Kutay, 2017). Despite this continuity, these membrane domains exhibit different composition and are subject to differential remodeling. In many fungi, the NE persists throughout both meiotic divisions, and tubulin and regulatory proteins are imported into the nucleus to control spindle assembly and dynamics. In *S. pombe*, while the NE is maintained along meiosis, the nucleocytoplasmic barrier is transiently lost at anaphase II (Arai et al., 2010; Asakawa et al., 2010). This process occurs without NPC disassembly but could rely on the modification of specific nucleoporins (Asakawa et al., 2016). This process allows timely nuclear release of proteins involved in ascospore formation (Yang et al., 2020), and facilitates spindle disassembly after meiosis II, likely by allowing access to the nucleus to spindle disassembly factors (Flor-Parra et al., 2018). Therefore, this remodeling process allows bidirectional nucleocytoplasmic transport of proteins whose localization is critical to conclude meiosis and facilitate the subsequent meiotic differentiation.

The NE also undergoes profound remodeling to promote meiotic cellular rejuvenation (King and Unal, 2020; Koch-Bojalad et al., 2021). In budding yeast, remodeling of the NE at meiosis II leads to the formation of a distinct NE compartment–the Gametogenesis Uninherited Nuclear Compartment (GUNC)–, which segregates senescence factors away from nuclei following meiosis (King et al., 2019). This compartment results from the division of the NE into five sub-compartments (GUNC and the four meiotic nuclei), and sequesters selected nuclear contents–like long-lived nucleoporins–and damaged material–including nucleolar and aggregated proteins–preventing their incorporation into ascospores (Fuchs and Loidl, 2004; King et al., 2019). GUNC formation depends on the ESCRT-III complex and on the formation of the prospore membrane (King et al., 2019; Koch et al., 2020), and is ultimately degraded following ascospore formation by Ndt80-dependent vacuole permeabilization (King et al., 2019). Consistent with a relevant role for this process in cell rejuvenation, the lifespan of the offspring of meiotic cells defective for GUNC formation is reduced (Koch et al., 2020). In *S. cerevisiae*, the ascospore INM proteome is distinct from that of mitotic cells, and it is mostly produced *de novo* following meiotic differentiation rather than being inherited from parental cells (Shelton et al., 2021). This regulated INM remodeling could be related to GUNC compartmentalization. However, this hypothesis remains to be tested.

In addition to the NE, the peripheral ER domains also undergo dynamic meiotic remodeling. Akin to mitochondria, regulated changes in ER arrangement and distribution facilitate its selective meiotic inheritance (Suda et al., 2007; Otto et al., 2021). During *S. cerevisiae* early meiosis, most cortical ER coalesces and is subsequently relocated to the cell central area by anaphase II–in a process called ER collapse–, to be ultimately segregated into ascospores. During this process, certain ER domains remain attached to plasma membrane by the tricalbins and Ist2 tethering proteins. Consequently, these domains are excluded from ascospores upon their formation, and are later degraded by vacuolar lysis (Otto et al., 2021). Like mitochondria, detachment of the ER from the plasma membrane depends on the transcriptional regulator Ndt80. In addition, ER detachment requires the reticulon and Yop1 proteins–which shape the ER by promoting membrane curvature–, as well as the lunapark protein Lnp1, which regulates ER network formation. ER collapse depends on actin dynamics and is followed by the elimination of a second subset of ER by selective ER autophagy (ER-phagy), which defines the ER to be segregated into ascospores (Otto et al., 2021). These findings revealed that meiotic inheritance of the ER relies on a sophisticated system that involves two parallel pathways for ER degradation, which selectively eliminate distinct ER subdomains, most probably as a quality control mechanism ensuring progeny rejuvenation. Further research should disclose the nature of the domains destined to degradation, as well as the mechanisms implicated in their recognition.

In *P. anserina*, as in budding yeast, the ER is subject to developmental remodeling during meiotic differentiation. In this fungus, ER subdomains enriched for the reticulon Rtn1 are differentially distributed during meiotic development. Rtn1 accumulates at the growing apical region of prophase I asci and relocates to the middle region during the subsequent meiotic progression. While cortical ER collapse has not been studied, Rtn1 is required for meiotic spindle arrangement and positioning, and its elimination leads to defective meiotic nucleus segregation (López-Fuentes et al., 2021). This suggests that in this fungus ER remodeling is linked to meiotic spindle dynamics. The precise function of Rtn1 in this process remains undisclosed. In budding yeast, loss of Rtn1 and Yop1 leads to defective mitotic spindle structure and positioning, resulting from alterations in SPB integrity (Casey et al., 2012). SPBs insert in the NE in regions of high membrane curvature, where the INM and ONM fuse (Jaspersen, 2021). Therefore, meiotic SPB assembly and integrity in *P*. *anserina* could rely on Rtn1. Consistent with this hypothesis, Rtn1 is also required for ascospore individualization in some asci, where it could facilitate SPB-driven prospore membrane formation (López-Fuentes et al., 2021). The processes of SPB and NPC insertion into the NE are interrelated and depend on common factors (Jaspersen and Ghosh, 2012), including the ER-shaping proteins (Dawson et al., 2009). Therefore, *P. anserina* Rtn1 could also be required for proper NPC function or remodeling during meiosis. In keeping with this hypothesis, a fraction of Rtn1 localizes to discrete puncta in the nuclear periphery during meiosis (López-Fuentes et al., 2021). Interestingly, the putative *S. macrospora* nucleoporin Pom33 interacts with ER-shaping proteins, including a reticulon protein (Groth et al., 2021). Although the location of this interaction is unknown, this could reflect a link between ER remodeling and the signaling pathways governing sexual development (Groth et al., 2021; Kuck and Stein, 2021).

Cortical ER remodeling could also be a determinant for meiotic nucleus segregation. In addition to mitochondria tethering, Num1 provides a cortical anchor for the microtubule motor dynein, which controls from the cell periphery the movement and positioning of nuclei and spindles, by pulling the astral microtubules emanating from the SPBs (Greenberg et al., 2018; Xiang, 2018). In *S. cerevisiae* mitotic cells, disruption of the cortical ER by deletion of the reticulon and Yop1 proteins alters Num1 cortical distribution (Lackner et al., 2013), while loss of the plasma membrane-ER tethering proteins Scs2/Scs22 perturbs Num1 localization and disturbs the microtubule-sliding activity of dynein (Omer et al., 2018). The meiosis cortically retained ER could be required to sustain spindle dynamics throughout meiosis. Under this scenario, *P. anserina* Rtn1 could be required for proper Num1-dependent localization of dynein during meiosis. Actually, the Num1 orthologue of *P. anserina* Ami1 is required for correct meiotic nuclear distribution and spindle positioning (Graia et al., 2000; Bouhouche et al., 2004). Nonetheless, while the association of mitochondria with Num1, and their role in dynein anchoring are conserved in yeasts; Num1 does not associate with the ER in *S. pombe* (Kraft and Lackner, 2019). Furthermore, Mdm36 is not conserved beyond Saccharomycotina. Further comparative analyses are required to better appreciate the participation of the ER in Num1 activity, as well as the specific and conserved roles performed by this protein, including its contribution to meiotic development. These analyses should enlighten the role of Num1 in integrating the dynamics of multiple membrane compartments with the cytoskeletal system driving nuclear segregation.

## Discussion

Recent research involving different model fungi has disclosed that meiotic development involves sophisticated regulation of the dynamics of numerous organelles, including peroxisomes, mitochondria and the ER. Furthermore, in addition to regulated nucleus dynamics–an intrinsic part of meiosis–the NE undergoes extensive remodeling supporting roles beyond chromosome compartmentalization.

**FIGURE 2:**
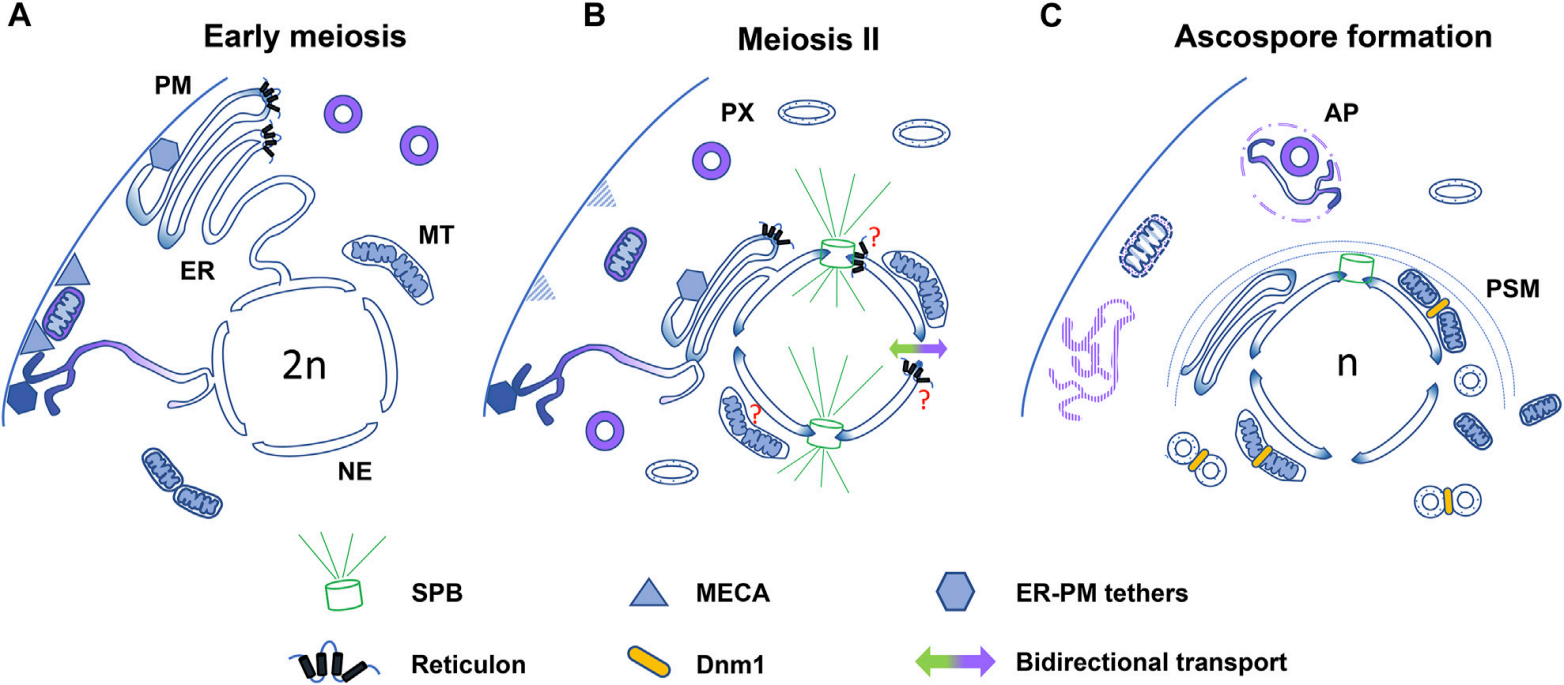
Model for organelle dynamics during fungal meiotic development. Diagram summarizing different processes involved in the regulation of organelle dynamics in different fungi. **(A)** In early meiosis, in *S. cerevisiae*, mitochondria (MT) and cortical ER are tethered to the plasma membrane (PM) through interactions with MECA (triangles) and different ER tethering proteins (hexagons with different shading), respectively. Increased mitochondrion fusion also takes place at this stage. In *P. anserina,* peroxisomes (PX) and reticulon (Rtn1)-rich ER domains exhibit a polarized localization. **(B)** Upon meiosis II (only one dividing nucleus is shown), in *S. cerevisiae* Ndt80-dependent transcriptional changes promote MECA degradation and ER-shaping protein-dependent detachment of the ER from the cell cortex (ER collapse). Mitochondria and most ER are relocated to the cell central area, while specific tethers retain a subset of ER at the cell cortex. In *P. anserina*, Rtn1 relocates to the cell central area following prophase I, where it could support SPB- and/or NPC-dependent spindle dynamics. During meiosis II, peroxisomes distribute more homogeneously and adopt a more elongated morphology than those of early meiosis, and they also differ in their protein import competency (illustrated by different shading). In *S. pombe*, the nucleocytoplasmic barrier is transiently lost at anaphase II (bidirectional arrow). **(C)** During ascospore formation, SPB-driven prospore membrane (PSM) formation conducts the packaging of meiotic nuclei, portions of the ER, nuclear-associated mitochondria and peroxisomes into nascent ascospores. In *S. cerevisiae*, the plasma membrane-retained ER is degraded upon vacuole permeabilization, and a second subset of the ER is eliminated by ER-phagy (AP, autophagosome). Dnm1-dependent fission is required for mitochondrion segregation. In *P. anserina*, Dnm1 is required for peroxisome segregation. Pexophagy could eliminate selected peroxisomes in *P. anserina* and in *S. macrospora*.

Organelle dynamics during meiotic development are regulated by membrane remodeling and fission and fusion processes, as well as by positional control systems, which define organelle arrangement, connectivity, and distribution. In addition, organelle proliferation and degradation modulate organelle abundance and selective removal during meiotic differentiation (Figure 2). The spatiotemporal coordination of these processes is important to promote balanced organelle segregation and defines quality control systems ensuring the inheritance of functional organelles, promoting progeny rejuvenation. Moreover, although many of their underlying involvements remain ambiguous, organelle dynamics are also critical for the regulation of cytoskeletal dynamics, which conduct accurate nuclear segregation, and for proper ascospore (gamete) differentiation.

The meiotic regulation of the dynamics of different organelles involves shared factors and common regulatory systems. Moreover, regulated interactions between distinct membrane compartments play fundamental roles in their dynamic regulation. These observations suggest commonality in many processes regulating organelle dynamics during meiotic development, and underscore close organelle interrelationships involved in orchestrating intracellular dynamics during this process. Still, not much is known beyond yeasts about the meiotic regulatory systems controlling organelle dynamics. Future comparative research should identify new such systems and disclose their evolutionary conservation. Further research should also increase our knowledge about the molecular mechanisms, inter-organelle crosstalk and developmental outcomes of organelle dynamics during meiotic development. Fundamental questions in this regard concern the identity of the organelles that are subject to differential remodeling in meiotic cells, as well as the mechanisms that conduct their selective transport, retention, and degradation, including the role of cytoskeletal and tethering proteins in these processes. High resolution and live-cell microscopy, along with interactome studies should provide significant insights into these issues. Further investigations should also disclose additional dynamic organelle remodeling processes and reveal whether equivalent processes are involved in meiotic regulation throughout diverse eukaryotes.

## Data Availability

The original contributions presented in the study are included in the article/Supplementary Material, further inquiries can be directed to the corresponding author.
